# Giant duodenal lipoma: an unusual cause of gastrointestinal bleeding (a case report)

**DOI:** 10.11604/pamj.2021.38.342.28951

**Published:** 2021-04-09

**Authors:** Marouane Baiss, Anwar Rahali, Hicham Elmajdoubi, Jalil Mdaghri, Khalid Lahlou, Rahal Mssrouri, Said Benamr, Abdellatif Settaf

**Affiliations:** 1 Department of Hepatobiliary, Digestive and Endocrine Surgery, Department of Surgery B, University Hospital Center Ibn Sina, Rabat, Morocco

**Keywords:** Lipoma, duodenum, melena, endoscopy, case report

## Abstract

Duodenal lipoma is a rare location of visceral lipomas, most are found incidentally via endoscopy or surgery and usually are asymptomatic. We report the case of a 58-year-old patient with an active bleeding duodenal lipoma. Although endoscopic treatment was scheduled initially, surgical intervention ultimately was indicated due to large size of tumor.

## Introduction

Lipomas of the duodenum are nonepithelial benign tumors of relatively uncommon findings; 64% of gastrointestinal lipomas are diagnosed in the colon, but only 4% seen in the duodenum [1]. It is a little known cause of gastrointestinal bleeding because they are usually asymptomatic. We describe a case of melena caused by a giant duodenal lipoma in a patient without any particular pathological history.

## Patient and observation

Mr MC, 58 years old, without any particular pathological history. For the past 3 months, he had intermittent episodes of melena and symptomatic anemia. A physical examination of his abdomen was normal. Laboratory test results revealed normocytic anemia (hemoglobin 7.8 g/dL). Esophagogastroduodenoscopy showed a smooth, soft, sessile polypoid lesion measuring around 10 cm in the bulb of the duodenum. At the most distal end of the mass, there was a Forrest type 2B ulcer that was oozing blood and remnant bloody material in the stomach were noted. Multiple biopsies taken from the ulcerated lesion and underlying polypoid lesion. No significant submucosal tissue or malignancy was present in histological analysis. Endoscopic ultrasonography showed a homogeneous and hyperechoic tumor in the submucosal layer of the duodenal wall, which was suggestive of a duodenal lipomatous mass. Given the size of the lesion and the bleeding character on contact, endoscopic polypectomy was practically impossible.

**Figure 1:**
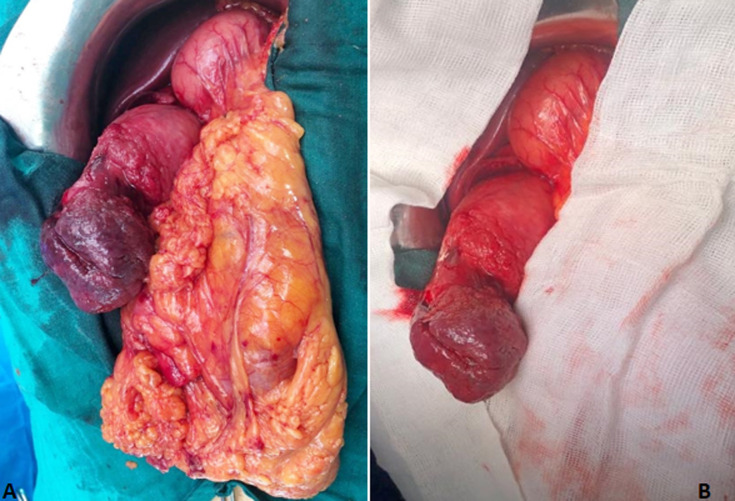
intraoperative images showing lipoma after duodenotomy

**Figure 2:**
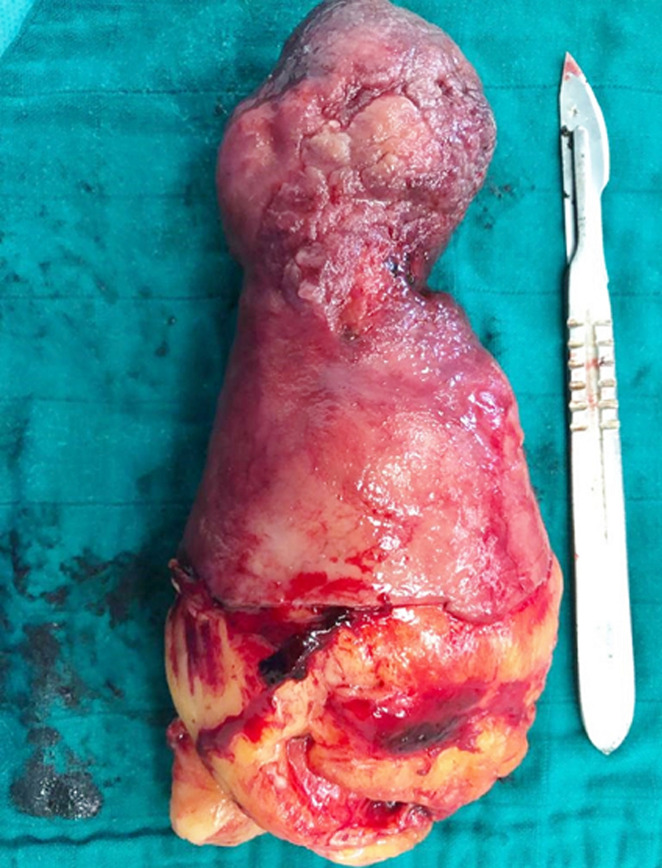
the giant duodenal lipoma after resection

The surgical indication was established. He underwent a surgical excision of the polyp by laparotomic approch. After 5 cm longitudinal incision of D2, a 10-cm polyp with ulceration of the tip was found protruding from the bulb of the duodenum (Figure 1). The polyp was extracted with ablation of its implantation base (Figure 2). Minutious dudenoplasty was done at the end of the surgical operation. The histological analysis of the resected lesion was in favor of lipoma without signs of malignancy. The postoperative surveillance was without abnormalities. Three months after the surgery, the patient presented no complications and no signs of gastrointestinal bleeding recurrence.

## Discussion

Lipomatous tomors of the gastrointestinal tract are rare and those of the duodenum are extremely unusual; 64% of gastrointestinal lipomas have been described in all parts of the colon, but only 4% occur in the duodenum. They are usually located in D2 where they arise from the submucosal adipose tissue in 95 % of cases [1,2]. The most of gastrointestinal (GI) lipomas are found incidentally via radiology, endoscopy, surgery or autopsy. However, large duodenal lipomas may present with clinical symptoms such as abdominal pain, dyspepsia, intussusception and rarely, GI hemorrhage and iron deficiency anemia due superficial ulcerations [3], as in our case. Esophagogastroduodenoscopy with biopsies is sufficient to orient the diagnosis of duodenal lipomas. But, the accurate positive diagnosis should only be made after tumor excision and histopathologic examinations to exclude specially the possibilities of malignancy. The main differential diagnosis is well-differentiated liposarcoma, pleomorphic lipoma and pleomorphic liposarcomas [4].

Current imaging means, such as computed tomography (CT) scan, magnetic resonance imaging (MRI) and endoscopic ultrasonography can give a correct diagnosis. Duodenal lipomas appear on CT as a well-circumscribed hypodense lesion with a fat density. In MRI, lipomas have high-intensity on T1 and usually present intermediate intensity on T2. Endoscopic ultrasonography is useful to dispense information about the original layer, echogenicity, the depth and invasion [5]. Symptomatic large lipomas should be removed endoscopically if standard endoscopic methods has not a high risk of perforation or bleeding specially with the endoloop-assisted unroofing technique. Indeed, the perfect application of an endoloop may prevent the risk of perforation or hemorrhage [6].

The surgical extirpation is indicated in sessile and large lesions because of the high risk of duodenal perforation. Operative management is mainly divided into: excision of the lipoma via a duodenotomy, segment bowel resection, pancreas-sparing duodenectomy or bypass. The type of procedure adopted to perform depends on the patient´s condition, the size and specially the localization of the lesion which is the key step. Most of the cases are managed by opening the duodenum and resected the mass as for our patient [6].

## Conclusion

Duodenal lipoma is a rare cause of gastrointestinal bleeding. The successful diagnostic means is esophagogastroduodenoscopy with biopsies. The endoscopic resection is the main treatment but many of these lesions need surgery as a result of their size and position.
